# Destabilization of MYC/MYCN by the mitochondrial inhibitors, metaiodobenzylguanidine, metformin and phenformin

**DOI:** 10.3892/ijmm.2013.1545

**Published:** 2013-11-01

**Authors:** STEPHANIE S. WANG, RUTH HSIAO, MARIKO M. LIMPAR, SARAH LOMAHAN, TUAN-ANH TRAN, NOLAN J. MALONEY, NAOHIKO IKEGAKI, XAO X. TANG

**Affiliations:** Department of Anatomy and Cell Biology, College of Medicine, University of Illinois at Chicago, Chicago, IL 60612, USA

**Keywords:** histone acetylation

## Abstract

In the present study, we investigated the anticancer effects of the mitochondrial inhibitors, metaiodobenzylguanidine (MIBG), metformin and phenformin. ^131^I-MIBG has been used for scintigraphic detection and the targeted radiotherapy of neuroblastoma (NB), a pediatric malignancy. Non-radiolabeled MIBG has been reported to be cytotoxic to NB cells *in vitro* and *in vivo*. However, the mechanisms behind its growth suppressive effects have not yet been fully elucidated. Metformin and phenformin are diabetes medications that are being considered in anticancer therapeutics. We investigated the anticancer mechanisms of action of MIBG and metformin in NB. Our data revealed that both drugs suppressed NB cell growth and that the combination drug treatment was more potent. MIBG reduced MYCN and MYC expression in *MYCN*-amplified and non-*MYCN*-amplified NB cells in a dose- and time-dependent manner. Metformin was less effective than MIBG in destabilizing MYC/MYCN. The treatment of NB cells with metformin or MIBG resulted in an increased expression of genes encoding biomarkers for favorable outcome in NB [(ephrin (*EFN*)*B2, EFNB3,* EPH receptor B6 (*EPHB6*)*,* neurotrophic tyrosine kinase, receptor, type 1 (*NTRK1*)*, CD44* and Myc-interacting zinc finger protein (*MIZ-1*)] and tumor suppressor genes [(early growth response 1 (*EGR1*)*,* EPH receptor A2 (*EPHA2*)*,* growth arrest and DNA-damage-inducible, beta (*GADD45B*)*,* neuregulin 1 (*NRG1*)*,* TP53 apoptosis effector (*PERP*) and sel-1 suppressor of lin-12-like (*C. elegans*) (*SEL1L*)]. Accordingly, metformin and MIBG augmented histone H3 acetylation in these cells. Phenformin also exhibited histone modification and was more effective than metformin in destabilizing MYC/MYCN in NB cells. Our data suggest that the destabilization of MYC/MYCN by MIBG, metformin and phenformin and their effects on histone modification are important mechanisms underlying their anticancer effects.

## Introduction

Neural crest-derived tumors characteristically express the norepinephrine transporter (NET). This feature can be used to specifically target tumor cells with agonists of NET. In this study, we investigated the anticancer effects and underlying mechanisms of action of mitochondrial inhibitors using neuroblastoma (NB), a pediatric tumor of neural crest origin, as an experimental system. NB is unique due to its clinical heterogeneity. While some tumors (favorable NB) are easily treatable, almost 50% of tumors (unfavorable NB) exhibit very aggressive behavior. Unfavorable NBs are also classified as high-risk NB and are characterized by widespread tumor dissemination, late relapse and poor long-term survival. Among the current treatments for high-risk NB, ^131^I-metaiodobenzylguanidine (MIBG) has been used for scintigraphic detection and the targeted radiotherapy of NB (1,2). The use of ^131^I-MIBG in clinical practice for the treatment of patients with NB is based on the fact that MIBG is a norepinephrine analogue and that NBs often express norepinephrine transporters or NET.

*MYC* and *MYCN* are proto-oncogenes that are members of the *MYC* gene family. A high MYCN/MYC expression is associated with the poorest disease outcome in NB. Approximately half of high-risk NBs exhibit *MYCN* amplification, which is associated with older age, rapid tumor progression and the poorest prognosis (3). A previous study suggested that in non-*MYCN*-amplified tumors, MYC expression is responsible for the aggressive phenotype (4). MYC and MYCN proteins are stemness factors whose expression, along with the expression of octamer-binding transcription factor 4 (OCT4), sex Determining Region Y)-box 2 (SOX2) and Kruppel-like factor 4 (KLF4), help somatic cells regain a stem cell phenotype (5). In a recent study, we suggested that the high expression of MYC/MYCN is critical to the existence of stem cell-like tumor-initiating NB cells (6). These observations suggest that MYCN and/or MYC expression are among the major determining factors of NB aggressiveness. Previously, we also found that mitochondrial inhibitors, such as carbonylcyanide p-trifluoromethoxyphenylhydrazone (FCCP), can destabilize MYC/MYCN and suppress the growth of NB cells (7), suggesting the potential use of mitochondrial inhibitors in the treatment of NB.

Studies on genes encoding biomarkers for favorable outcome in NB (favorable NB genes) have revealed mechanisms underlying the favorable phenotype of the tumor. To date, several favorable NB genes have been identified, and include [EPH receptor B6 (*EPHB6*)*,* ephrin (*EFN)B2, EFNB3,* neurotrophic tyrosine kinase, receptor, type 1 (*NTRK1; TrkA*), *CD44* and Myc-interacting zinc finger protein (*MIZ-1*)] (8–13). High expression levels of favorable NB genes are associated with a favorable outcome in NB, and the enforced expression of these genes in cells in unfavorable NB results in growth suppression. We previously reported that known favorable NB genes are epigenetically silenced in cells associated with an unfavorable outcome in NB (9,14). In the present study, we investigated the effects of three additional mitochondrial inhibitors, MIBG, metformin and phenformin on MYC/MYCN. Metformin and phenformin are diabetes medications that are currently being considered for use as anticancer drugs. Our data suggest that the destabilization of MYC/MYCN by MIBG, metformin and phenformin is key to their antitumor effects. In addition, the effects of these drugs on histone modification, which is at least in part responsible for a change in the global gene expression pattern of cells (i.e., upregulated expression of favorable NB genes and tumor suppressor genes), may provide another mechanism underlying their anticancer effects.

## Materials and methods

### Reagents and drug treatments

MIBG and metformin were purchased from Calbiochem/EMD Chemicals, San Diego, CA, USA. MIBG was dissolved in 5 mM hydrochloric acid (HCl) at the concentration of 10 mM as stock solution. Metformin was dissolved in H_2_O at the concentration of 0.5 M as stock solution. Phenformin was purchased from Sigma-Aldrich (St. Louis, MO, USA), and the stock solution was made in 5 mM HCl at 0.25 M. The medium containing fresh drugs was changed every 48 h.

### Cell lines and western blot analysis

SKNBE(2)C, IMR5, Nb69, SY5Y and SKNAS cells were cultured as previously described (6). SKNBE(2)C and SY5Y cells were provided by Dr Robert Ross (Fordham University, Bronx, NY, USA). IMR5 and Nb69 cells were from Dr Roger Kennett and Dr Fred Gilbert (University of Pennsylvania, Philadelphia, PA, USA). SKNAS cells were from Dr C. Patrick Reynolds (The Texas Tech University Health Sciences Center, Lubbock, TX, USA). Western blot analysis was performed as previously described (6). MYC and MYCN were detected using the mouse monoclonal antibodies, NCM II 143 and NCM II 100 (15), respectively.

### Reverse transcription and TaqMan real-time polymerase chain reaction (PCR)

RNA was isolated using the Qiagen RNeasy kit (Qiagen, Valencia, CA, USA). Two micrograms of total RNA were used to synthesize cDNA. Reverse transcription and TaqMan real-time PCR were performed as previously described (6).

### 3-(4,5-Dimethylthiazol-2-yl)-5-(3-carboxymethoxyphenyl)-2-(4-sulfophenyl)-2H-tetrazolium, inner salt (MTS) assay

An MTS assay (Promega, Madison, WI, USA) was performed as described in our previous study (6).

## Results

### Growth suppressive effect of MIBG and its ability to destabilize MYC/MYCN in NB cells

**Figure 1:**
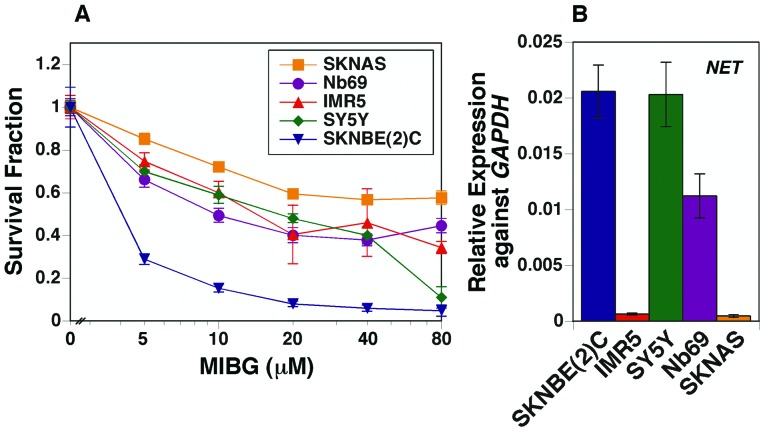
Growth suppressive effect of metaiodobenzylguanidine (MIBG) and the expression of norepinephrine transporter (NET) in neuroblastoma (NB) cells. (A) The NB cell lines indicated were treated with various concentrations of MIBG (shown as log scale at the x-axis) for 4 days. MTS assay was employed to assess the growth suppressive effects of MIBG on these cells. (B) NET expression in the indicated NB cell lines was examined in duplicate by TaqMan real-time polymerase chain reaction (PCR) using gene-specific TaqMan gene expression assays.

Non-radiolabeled MIBG has been reported to be cytotoxic to NB cells *in vitro* and *in vivo*(16). However, the mechanisms underlying its growth suppressive effects are not yet well understood. In this study, we examined the growth suppressive effects of MIBG on 5 NB cell lines and investigated the possible mechanisms responsible for its effects. MIBG suppressed the growth of *MYCN*-amplified NB cells [SKNBE(2)C and IMR5 cells] and non-*MYCN*-amplified NB cells (SKNAS, Nb69 and SY5Y cells) (Fig. 1A). In addition, SKNBE(2)C cells were the most susceptible to the effects of MIBG. Fig. 1B shows the NET expression data in the cell lines examined. SKNAS cells expressed the lowest levels of NET among the cell lines examined, which was consistent with the observation that MIBG had the least potent effect in suppressing the growth of SKNAS cells. Nonetheless, there was no direct correlation between NET expression and growth suppression mediated by MIBG in the other four cell lines (Fig. 1B).

**Figure 2:**
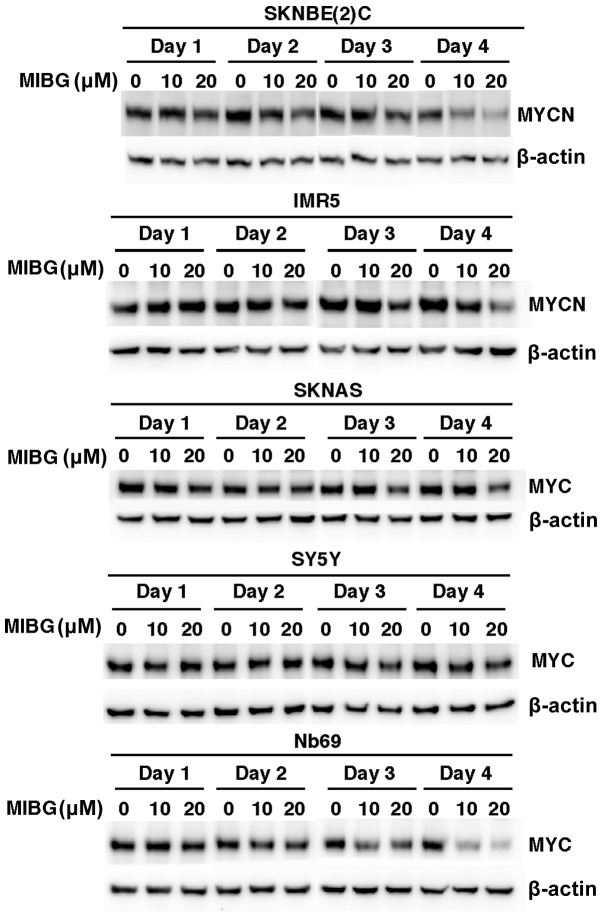
Metaiodobenzylguanidine (MIBG) destabilizes MYCN and MYC in neuroblastoma (NB) cells in a dose- and time-dependent manner. NB cells indicated were treated with MIBG (10 and 20 μM) for different periods of time, as indicated. The cells were subsequently subjected to western blot analysis using the MYCN-specific antibody, NCM II 100, for SKNBE(2)C and IMR5 cells or the pan-MYC reactive mouse monoclonal antibody, NCM II 143, for the SKNAS, SY5Y and Nb69 cell lines. Total protein (5 μg) was loaded per lane. Control untreated SKNBE(2)C and IMR5 cells expressed high levels of MYCN, whereas the control untreated SKNAS, SY5Y and Nb69 cells expressed high levels of MYC.

We previously demonstrated that FCCP, a known mitochondrial inhibitor, destabilized MYC and MYCN in NB cells and induced a growth suppressive effect (7). In this study, to investigate whether MIBG, which is also a mitochondrial inhibitor, exhibits a similar effect on MYC/MYCN in NB cells, we examined MYC/MYCN expression in NB cell lines treated with MIBG (10 and 20 μM) for 4 days. SKNBE(2)C and IMR5 cells expressed high levels of MYCN, whereas SKNAS, SY5Y and Nb69 cells expressed high levels of MYC. Western blot analysis revealed that the treatment of these NB cell lines with MIBG resulted in a reduction in MYCN or MYC expression in a time-dependent manner (Fig. 2). The decreased MYC/MYCN expression was detected as early as day 2 [SKNBE(2)C cells] and day 3 (IMR5, SKNAS and Nb69 cells) of the drug treatment. The destabilization of MYC/MYCN by MIBG also occurred in a dose-dependent manner in SKNBE(2)C, IMR5, SKNAS and Nb69 cells. Unlike the other cell lines, the MIBG-treated SY5Y cells showed only a 15% reduction in MYC expression on day 4 of the drug-treatment and at the dose of 20 μM (Fig. 2). The effects of MIBG on MYC/MYCN expression were most prominent in the SKNBE(2)C and Nb69 cells, followed by the IMR5 cells and, lastly, the SKNAS and SY5Y cells. Taken together, the data on the growth suppressive effects of MIBG, shown in Fig. 1A, and the effects of MIBG on MYC/MYCN, shown in Fig. 2, indicate that with the exception of SY5Y cells, there was a correlation between the growth suppressive effects of MIBG at 10 and 20 μM and its effects on MYC and MYCN expression among the other 4 NB cell lines. This observation suggests that the destabilization of MYC and MYCN is among the important mechanisms through which MIBG exerts its growth suppressive effects on NB cells.

### Effect of metformin as a single agent or in combination with MIBG on MYC/MYCN expression and growth of NB cells

**Figure 3:**
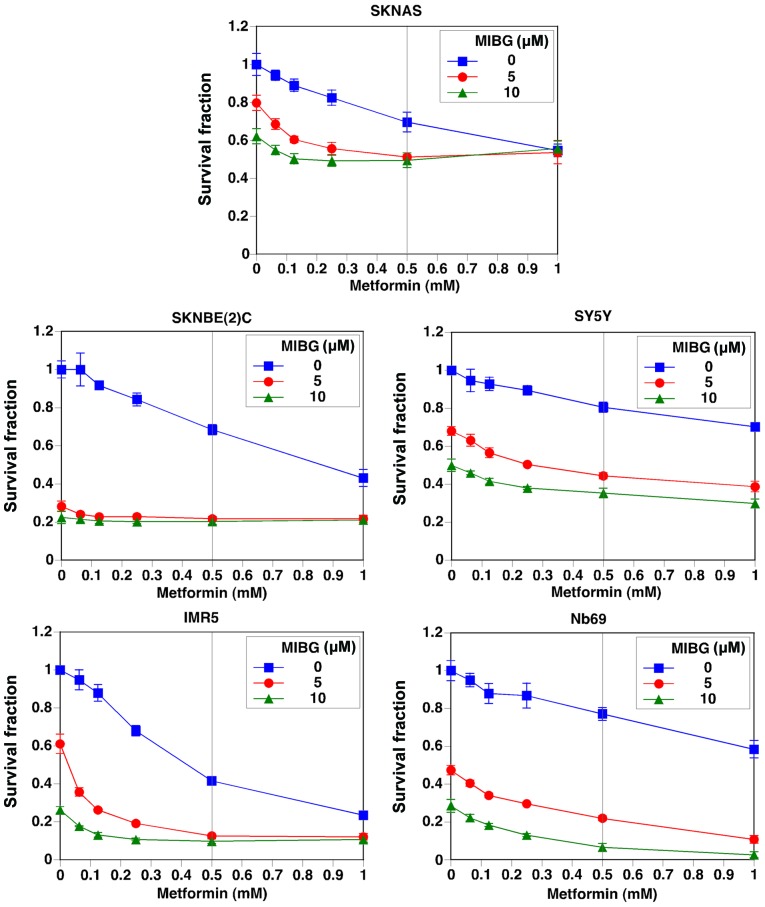
Growth suppressive effect of metaiodobenzylguanidine (MIBG) and metformin as single agents and in combination. The neuroblastoma (NB) cell lines indicated were treated with combinations of MIBG (5 or 10 μM) and metformin at various concentrations (0.1 to 1 mM) for 4 days. MTS assay was employed to assess the growth suppressive effects of the drugs.

To determine whether the growth suppressive effect and the MYC/MYCN destabilizing effect are general features of mitochondrial inhibitors, we extended our investigation to the effects of metformin on MYC/MYCN expression and the growth of NB cells. Although metformin is currently being tested in clinical trials as an anticancer drug, the mechanisms underlying its anticancer effects are not yet fully understood. The curve with square data points represents the effects of metformin as a single agent on NB cell growth, whereas the curves with a solid circle or solid triangle data points represent the effects of metformin in combination with MIBG at 5 and 10 μM, respectively (Fig. 3). Our results revealed that metformin as a single agent suppressed NB cell growth, and there was an additive growth suppressive effect by the combination treatment. Among the cell lines examined, the combination treatment was most effective in suppressing the growth of Nb69, IMR5 and SKNBE(2)C cells. The growth suppressive effect of metformin as a single agent was most effective in IMR5 cells and the least effective in SY5Y cells. Although the effects of metformin on cell growth were moderate in Nb69 cells, the effects were markedly enhanced when tested in combination with MIBG. The growth of SKNAS and SY5Y cells was the least affected under all treatment conditions.

**Figure 4:**
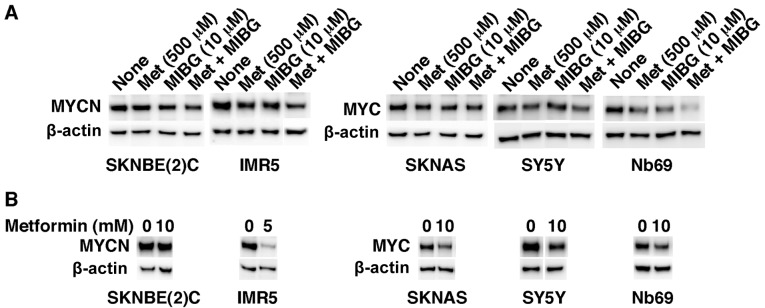
Reduction of MYCN/MYC expression in neuroblastoma (NB) cells following treatment with MIBG and metformin. The NB cell lines indicated were treated with (A) metformin (500 μM) and MIBG (10 μM) as single agents or in combination for 4 days, and (B) metformin (5 or 10 mM) as a single agent for 1 day. Western blot analysis was used to assess the MYC or MYCN levels as described in Fig. 2. Met, metformin.

We then investigated the effects of metformin (500 μM) as a single agent and in combination with MIBG (10 μM) on MYC/MYCN expression in NB cells. The reduction in MYC and MYCN expression in the cells treated with both drugs at the above concentrations was the greatest in Nb69 cells followed by IMR5 cells (Fig. 4A). In addition, the combination drug treatment was the most effective in destabilizing MYC/MYCN in the treated NB cells (Fig. 4A). The 2 drugs had the least MYC/MYCN destabilizing effect in SKNBE(2)C, SKNAS and SY5Y cells. Nonetheless, metformin at much higher concentrations (5 and 10 mM) was effective in reducing MYC/MYCN expression not only in IMR5 and Nb69 cells, as expected, but also in SKNAS and SY5Y cells after 1 day of the drug treatment (Fig. 4B). By contrast, SKNBE(2)C cells were resistant to metformin as regards the destabilization of MYCN even at the higher dose of the drug (Fig. 4B). The results presented in Fig. 4A correlate well with the data points corresponding to the growth suppressive effects of metformin (500 μM) and MIBG (10 μM) shown in Fig. 3 (see the indicative vertical line at 500 μM of metformin in Fig. 3). This observation further supports the hypothesis that the destabilization of MYC/MYCN is an important mechanism responsible for the ability of the drugs to suppress NB cell growth.

### Treatment of NB cells with metformin or MIBG results in increased expression of genes encoding biomarkers of favorable outcome in NB (EFNB2, EFNB3, EPHB6, NTRK1, CD44 and MIZ-1) and tumor suppressor genes [early growth response 1 (EGR1), EPH receptor A2 (EPHA2), growth arrest and DNA-damage-inducible, beta (GADD45B), neuregulin 1 (NRG1), TP53 apoptosis effector (PERP) and sel-1 suppressor of lin-12-like (C. elegans) (SEL1L)]

**Figure 5:**
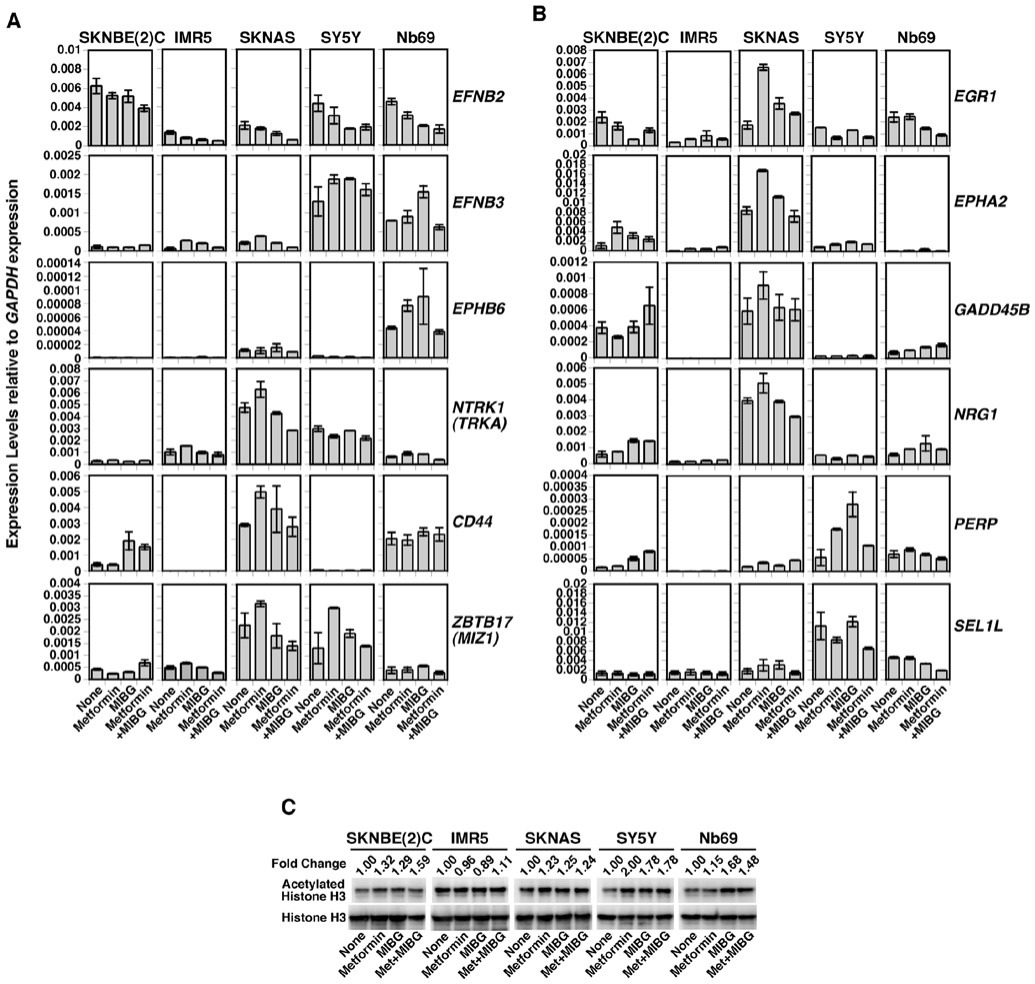
Effects of metformin and metaiodobenzylguanidine (MIBG) as single agents and in combination on (A) the expression of genes encoding biomarkers of favorable outcome in neuroblastoma (NB) and (B) tumor suppressor genes. The NB cell lines indicated were treated with metformin (500 μM) and/or MIBG (10 μM) for 4 days. The expression of the genes of interest was examined in duplicate by TaqMan real-time polymerase chain reaction (PCR) using gene-specific TaqMan gene expression assays. (C) Elevated expression of acetylated histone H3 in NB cell lines treated with metformin and/or MIBG. The NB cell lines indicated were treated with metformin (500 μM) and/or MIBG (10 μM) for 4 days. Western blot analysis was used to assess the expression levels of acetylated histone H3 and total histone H3 in the drug-treated cells and the untreated control cells. Fold change in acetylated histone H3 expression based on the densitometry analysis against histone H3 expression is shown. Met, metformin

The data shown in Figs. 2 and 3 suggest that in addition to the destabilization of MYC/MYCN, other mechanisms may be involved in the growth suppressive effects on NB cells mediated by MIBG and metformin, particularly in SY5Y and SKNAS cells. To explore the possibility that such mechanisms exist, we examined the effects of MIBG and metformin as single agents and in combination on the expression of genes encoding biomarkers of favorable outcome in NB (*EFNB2, EFNB3, EPHB6, NTRK1, CD44* and *MIZ-1*) in NB cells. The expression of tumor suppressor genes was also examined in the NB cell lines treated with MIBG and/or metformin. These genes included *EGR1, EPHA2, GADD45B, NRG1, PERP* and *SEL1L*. Metformin and MIBG upregulated the expression of favorable NB genes and that of tumor suppressor genes in the SKNBE(2)C, SKNAS, SY5Y and Nb69 cells (Fig. 5A and B). The drug treatments had little effect on the expression of these genes in the IMR5 cells.

### Metformin and MIBG increase the expression of acetylated histone H3

We previously demonstrated that the low expression of favorable NB genes (*EFNB2, EFNB3, EPHB6, NTRK1, CD44* and *MIZ-1*), as well as that of the tumor growth suppressive gene, *EPHA2,* in NB cell lines was due to epigenetic silencing (9,14,17). We thus examined whether metformin and MIBG have an effect on chromatin structure, mainly the alteration of the histone acetylation status, thereby leading to the increased expression of these genes. With the exception of IMR5 cells, the other NB cell lines treated with metformin and/or MIBG demonstrated an increased expression of acetylated histone H3 compared with the untreated control (Fig. 5C). These data suggest that metformin and MIBG function as histone deacetylase (HDAC) inhibitors, which in turn upregulates the expression of favorable NB genes and tumor suppressor genes. The inability of the drugs to augment acetylated histone H3 expression in the IMR5 cells is consistent with the data shown in Fig. 5A and B, which show that the drugs had little effect on the expression of the genes examined in IMR5 cells.

### Effect of phenformin on MYC/MYCN expression, acetylation of histone H3 and growth of NB cells

**Figure 6:**
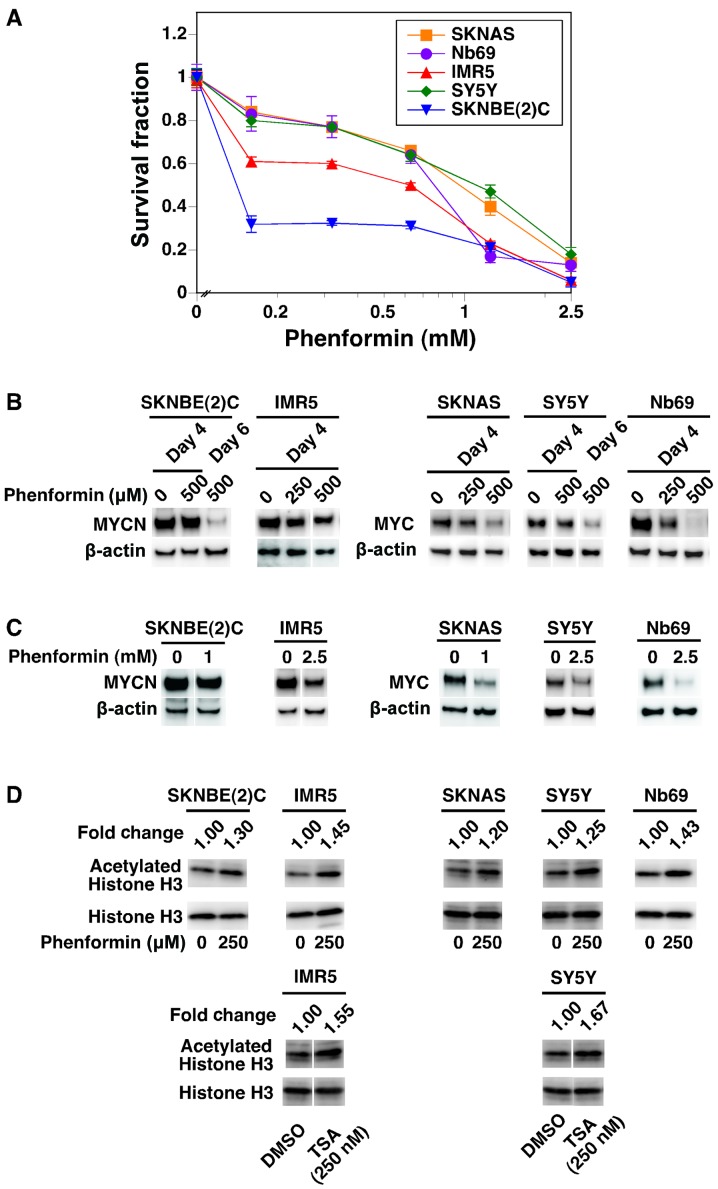
Effects of phenformin on MYC/MYCN expression, acetylation of histone H3 and growth of neuroblastoma (NB) cells. (A) The NB cell lines indicated were treated with phenformin at doses ranging from 0 to 2.5 mM (shown as log scale at the X-axis) for 48 h. MTS assay was employed to assess the growth suppressive effects of phenformin on these cells. (B) The NB cell lines indicated were treated with phenformin (250 and 500 μM) for 4 and 6 days, and (C) phenformin (1 and 2.5 mM) for 1 day. Western blot analysis was used to assess MYC or MYCN levels as described in Fig. 2. (D) The NB cell lines indicated were treated with phenformin (250 μM) for 4 days. Western blot analysis was used to assess the expression levels of acetylated histone H3 and total histone H3 in the drug-treated cells and the untreated control cells. IMR5 and SY5Y cells treated for 24 h with DMSO or 250 nM trichostatin A (TSA), a potent histone deacetylase inhibitor, were included as the controls. Fold change in acetylated histone H3 expression based on the densitometry analysis against histone H3 expression is shown.

Metformin at 500 μM (Fig. 4A) was less effective than MIBG at 20 μM (Fig. 2) in reducing MYC/MYCN expression in the NB cells. In addition, high concentrations of metformin were required to effectively reduce MYC/MYCN expression in the NB cells (Fig. 4B). We thus examined the effects of phenformin, another mitochondrial inhibitor and anti-diabetic drug, on MYC/MYCN expression in the NB cells. It has been reported that phenformin binds NET (18), suggesting that NET-positive cells, such as NB cells can preferentially uptake phenformin. As shown in Fig. 6A, phenformin induced growth suppressive effects on NB cells in a dose-dependent manner and destabilized MYC/MYCN at the dose of 250 or 500 μM on days 4 and 6 of the drug treatments (Fig. 6B). Phenformin was therefore more effective than metformin in reducing MYC/MYCN expression. Short-term and high-dose treatments of phenformin (1 day-treatment at the doses of 1 and 2.5 mM) destabilized MYC/MYCN (Fig. 6C). In addition, Fig. 6B and C show that phenformin destabilized MYCN more effectively in the low-dose/long-term treatment of SKNBE(2)C cells. Finally, the treatment of NB cells with phenformin resulted in an increased expression of acetylated histone H3 (Fig. 6D).

## Discussion

We investigated the anticancer effects and underlying mechanisms of action of mitochondrial inhibitors (MIBG, metformin, and phenformin) using NB cell lines as an experimental system. MIBG was previously known as a neuroendocrine tumor-targeting agent. ^131^I-MIBG has been used for scintigraphic detection and the targeted radiotherapy of NB (1,2). Historically, the radioactive^131^I residue on ^131^I-MIBG has been considered to be the therapeutic effector due to its radiotoxicity to NB. Our data demonstrated that non-radiolabeled MIBG confers a growth suppressive effect on NB cells, destabilizes MYC/MYCN and induces changes in global gene expression. The latter effect is partly due to the ability of MIBG to increase the acetylation of histone H3 in the cells.

Metformin and phenformin are anti-diabetic biguanides that reduce blood glucose levels by inhibiting gluconeogenesis in the liver. Metformin is one of the first-line medications for type II diabetes in the United States and other countries, while there is continued use of phenformin in certain European and South American countries. Epidemiological evidence suggests that metformin reduces cancer incidence and mortality in patients with breast and prostate carcinoma (19–21); however, its exact biochemical mechanisms are not yet well understood. There are two pre-existing ideas that need to be re-evaluated in order to gain better insight into the mechanisms through which biguanides exert their anticancer effects: i) the involvement of AMP-activated protein kinase (AMPK) in metformin function (22); and ii) the Warburg hypothesis (23), which states that cancer tissues are characterized by their enhanced glycolysis in oxidative conditions and impaired mitochondrial oxidative phosphorylation (OXPHOS) functions.

First, the results of several studies are inconsistent with the hypothesis that the anti-diabetic and growth inhibitory effects of metformin are linked to the activation of AMPK: i) studies using AMPK knockout mice have demonstrated that metformin inhibits mitochondrial OXPHOS Complex I and induces changes in the energy state, which is solely responsible for the inhibition of gluconeogenesis (24); ii) it has been suggested that the growth inhibitory effects of metformin are due to AMPK activation, leading to the inhibition of the mTOR pathway (25). Nonetheless, recent evidence has indicated that without functional AMPK, metformin upregulates *REDD1* transcripts and protein, which is involved in the negative regulation of the mTOR pathway (26). Thus, AMPK activation may be an epiphenomenon to the direct effects of metformin (mitochondrial OXPHOS inhibition and transcriptional activation).

Furthermore, several lines of evidence refute the Warburg hypothesis: i) Weinhouse’s re-evaluation of Warburg’s data indicated that tumor tissues are in fact active in mitochondria-driven OXPHOS (27); ii) several types of cancer cells express high levels of MYC family proteins that stimulate mitochondrial biogenesis, glycolysis and glutaminolysis, thus facilitating cancer cell growth (28). These observations suggest that cancer cells are dependent on mitochondrial OXPHOS augmented by elevated MYC expression. Thus, the inhibition of mitochondrial OXPHOS may have a profound effect on cancer cell growth. Our data revealed that mitochondrial inhibitors induce growth suppressive effects on NB cells *in vitro*. We also found that metformin and phenformin destabilize MYC and MYCN in NB cell lines, although their effective doses differ (see below).

Finally, the anti-diabetic biguanides interact with heavy metals (e.g., Zn, Cu and Fe) (29), which can affect the activities of Zn-dependent histone deacetylases. Our data (Figs. 5C and 6D) are consistent with this observation. The treatment of NB cells with metformin and phenformin resulted in an increase in the expression of acetylated histone H3. It remains to be seen whether the anti-diabetic biguanides have any effect on histone demethylase.

Our data suggest that the destabilization of MYC and MYCN is among the important mechanisms through which MIBG, metformin and phenformin exert their growth suppressive effects on NB cells. MIBG is more effective than metformin in destabilizing MYC/MYCN function, and phenformin is more effective than metformin, but less effective than MIBG in destabilizing MYC/MYCN in NB cell lines. Structurally, phenformin can be considered a hybrid molecule between MIBG and metformin. This may account for the observation that its anti-MYC/MYCN effects are not as effective as those of MIBG, but more effective than those of metformin. In addition, the mechanisms through which NB cells uptake drugs may, in part, explain the difference in the potency of the drugs in destabilizing MYC/MYCN. Among the 3 drugs examined, it was found that NB cells uptake MIBG via a receptor-mediated process due to the expression of NET on their surface. Phenformin may use a similar mechanism as MIBG since it has been shown to bind NET (18). Phenformin can also easily diffuse into the cells due to the presence of the phenol ring.

The two new biological and chemical functions of MIBG, metformin and phenformin reported in this study (specifically, the destabilizing of MYC/MYCN and the ability to augment the acetylation of histone H3) provide a better understanding of the mechanisms through which these drugs function. Phenformin binds NET, which is also expressed on the NB cell surface. Therefore, MIBG and phenformin can be tumor-targeting MYC/MYCN destabilizing agents in NB. The *in vitro* doses of metformin and phenformin used in this study seem relatively high. However, the effective doses *in vivo* (clinically) are lower than those used for our *in vitro* experiments. This is due to the fact that the positive charge of metformin and phenformin causes them to accumulate to a very high concentration in the negatively charged mitochondrial matrix over time (30). In a recent study, we suggested that MYC/MYCN are important stemness factors that play key roles during the development of NB stem cells or stem-like cells (6). Thus, the destabilization of MYC/MYCN by metformin and phenformin and the ability of metformin to upregulate tumor suppressor genes may partly explain why these diabetes drugs protect against cancer incidence and mortality.
